# Crystal structure and Hirshfeld surface analysis of 4-bromo-6-(4-chloro­phen­yl)-6,7-di­hydro-5*H*-furo[2,3-*f*]isoindol-5-one

**DOI:** 10.1107/S2056989025008606

**Published:** 2025-10-07

**Authors:** Kseniia A. Alekseeva, Atash V. Gurbanov, Mikhail S. Grigoriev, Elena A. Sorokina, Irina A. Kolesnik, Mohammed Hadi Al-Douh, Tuncer Hökelek, Khudayar I. Hasanov

**Affiliations:** a RUDN University, 6 Miklukho-Maklaya St., Moscow 117198, Russian Federation; b Excellence Center, Baku State University, Z. Khalilov Str. 33, AZ 1148, Baku, Azerbaijan; c Frumkin Institute of Physical Chemistry and Electrochemistry, Russian academy of Sciences, Leninsky prosp. 31, Build. 4, Moscow 119071, Russian Federation; d Institute of Physical Organic Chemistry, National Academy of Sciences of Belarus, Surganov Str. 13, Minsk 220072, Belarus; e Chemistry Department, Faculty of Science, Hadhramout University, Mukalla, Hadhramout, Yemen; f Hacettepe University, Department of Physics, 06800 Beytepe-Ankara, Türkiye; g Azerbaijan Medical University, Scientific Research Centre (SRC), A. Kasumzade Str. 14, AZ 1022, Baku, Azerbaijan; Texas A & M University, USA

**Keywords:** crystal structure, non-covalent inter­actions, iso­indole, IMDAV furo[2,3-*f*]iso­indole

## Abstract

The mol­ecule of the title compound, C_16_H_9_BrClNO_2_, contains furan and phenyl rings and an iso­indole ring system. The phenyl ring subtends a dihedral angle of 10.3 (2)° with the fused ring system. In the crystal, C—H⋯O hydrogen bonds link the mol­ecules into a two-dimensional network nearly parallel to the *ab* plane, enclosing *R*^2^_2_(6), *R*^4^_4_(12), *R*^4^_4_(14), *R*^4^_4_(18) and *R*^4^_4_(20) ring motifs. π–π stacking between the centroids of parallel rings [centroid–centroid distances = 3.919 (3)–3.695 (3) Å] helps to consolidate the packing. Hirshfeld surface analysis revealed that the most important contributions for the crystal packing are from H⋯H (21.2%), H⋯Cl/Cl⋯H (14.7%), H⋯O/O⋯H (13.9%), H⋯C/C⋯H (13.1%), H⋯Br/Br⋯H (12.3%) and C⋯C (11.6%) inter­actions.

## Abstract 2

The mol­ecule of the title compound, C_16_H_9_BrClNO_2_, contains furan and phenyl rings and an almost planar iso­indole ring system, which is coplanar with the furan ring. In the crystal, C—H⋯O hydrogen bonds link the mol­ecules into a two-dimensional network. π–π stacking helps to consolidate the packing.

## 1. Chemical context

Iso­indoles have emerged as a significant class of heterocyclic compounds in organic chemistry due to their diverse bio­logical activities as well as their utility in material science and organocatalysis (for recent reviews, see: Chen & Zou, 2021 ▸; Samandram *et al.*, 2025 ▸). Despite their importance, an efficient strategy for their synthesis remains to be found. Building on our and other studies of intra­molecular Diels–Alder reactions of vinyl­arenes (IMDAV) (Zaytsev *et al.*, 2021 ▸; Krishna *et al.*, 2022 ▸), we now investigate how 3-(ar­yl)allyl­amines undergo IMDAV reactions with halogenated maleic anhydride (Alekseeva *et al.*, 2020 ▸). As well as hydrogen bonds (Burkin *et al.*, 2024 ▸; Maharramov *et al.*, 2010 ▸, 2011 ▸; Pronina *et al.*, 2024 ▸), inter­molecular halogen bonds can also be used in the supra­molecular assembly of organic and coordination compounds and improve their functional properties (Gurbanov *et al.*, 2022 ▸; Shixaliyev *et al.*, 2013 ▸, 2014 ▸). We have recently reported a new synthetic strategy for constructing a condensed iso­indole scaffold, arising from a cascade transformation between 3-(2-fur­yl)allyl­aniline and di­bromo­maleic anhydride (Alekseeva *et al.*, 2025 ▸). Remarkably, substitution on the benzene ring of the starting aniline leads to a decrease in product yield, yet the overall reaction pathway remains unaffected, proceeding through deca­rboxylation and de­hydro­bromination. The resulting 6,7-di­hydro-5*H*-furo[2,3-*f*]-isoindol-5-ones represent versatile inter­mediates that can be further transformed to other iso­indole derivatives through Heck or Suzuki cross-coupling reactions, thereby providing a valuable entry into a broader class of functionalized iso­indole derivatives (Bartolucci *et al.*, 2012 ▸; Kalari *et al.*, 2017 ▸; Alzweiri *et al.*, 2021 ▸; Kumar *et al.*, 2023 ▸). Herein, we report the synthesis and mol­ecular and crystal structure of the title compound, **1**, together with a Hirshfeld surface analysis.
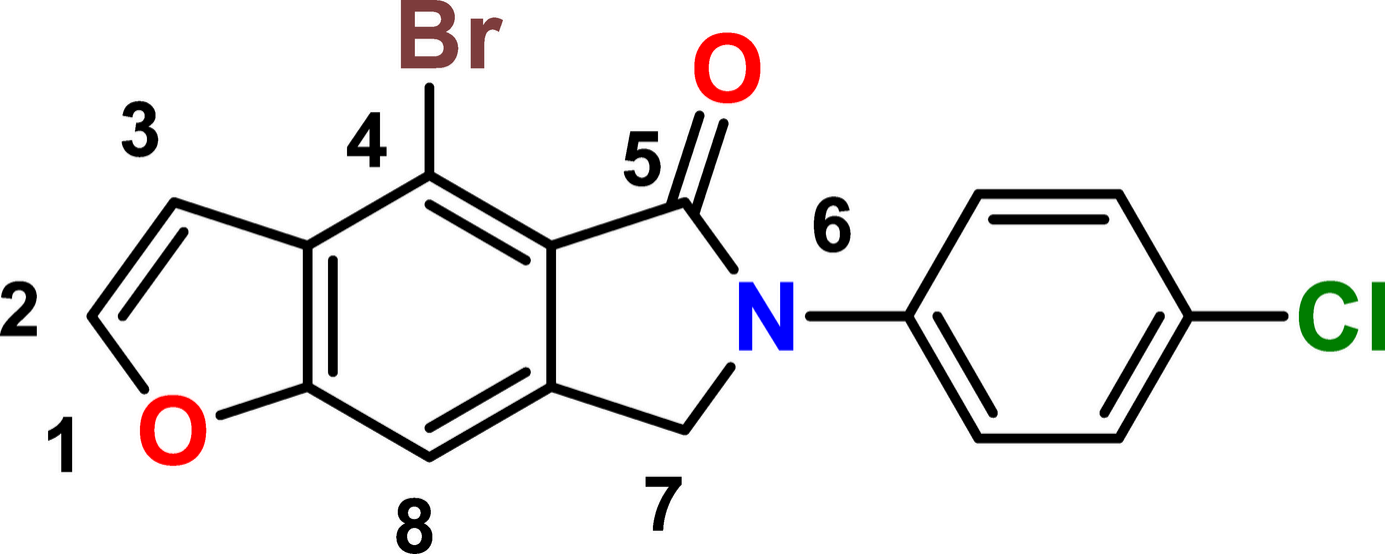


## 2. Structural commentary

**Figure 1:**
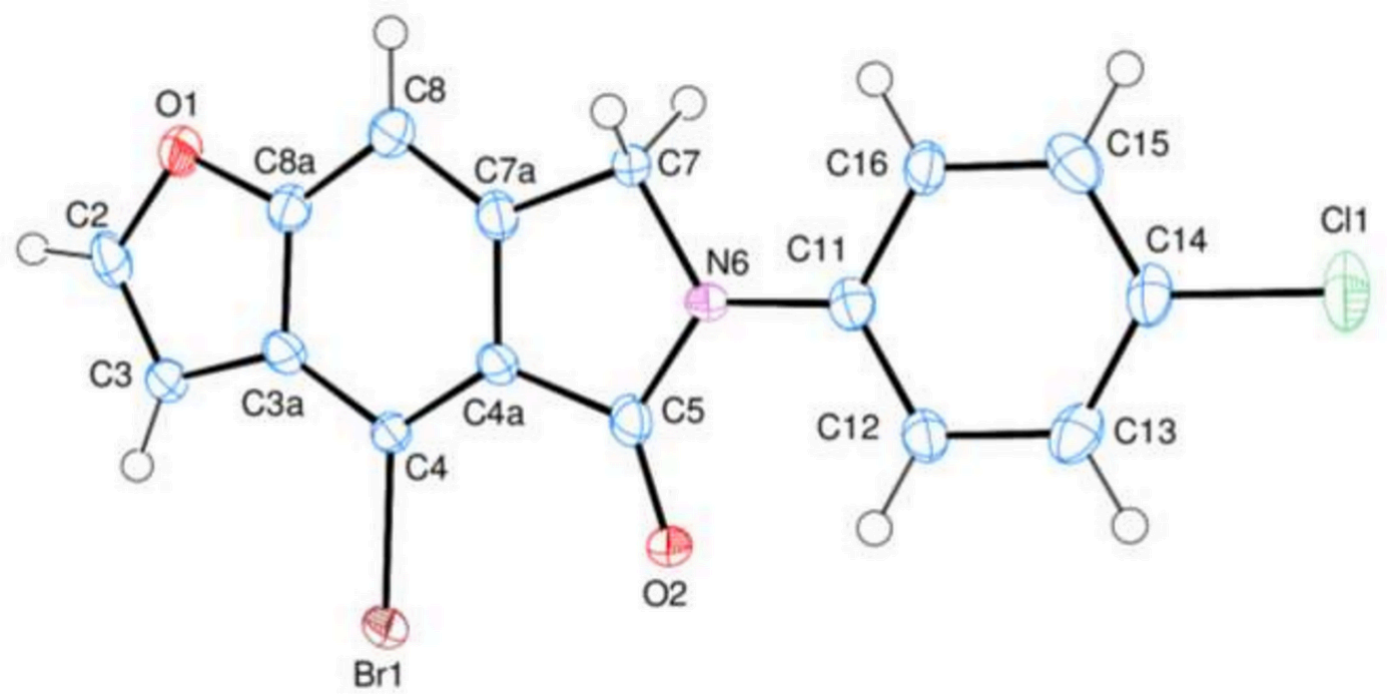
The asymmetric unit of the title compound with the atom-numbering scheme and 50% probability ellipsoids.

The asymmetric unit contains one mol­ecule comprising a pyrrole ring fused to a benzo­furan ring system, and a phenyl ring with substitutions on each one of them (Fig. 1 ▸). The individually planar rings *A* (O1/C2/C3/C3*A*/C8*A*), *B* (C3*A*/C4/C4*A*/C7*A*/C8/C8*A*) and *C* (C4*A*/C5/N6/C7/C7*A*), which are fused, and *D* (C11–C16) are oriented at dihedral angles of *A*/*B* = 0.9 (3)°, *A*/*C* = 1.8 (3)°, *A*/*D* = 10.5 (3)°, *B*/*C* = 0.9 (3)°, *B*/*D* = 10.0 (3)° and *C*/*D* = 9.9 (3)°. Thus, the *A*, *B* and *C* rings are essentially coplanar. The substituent atoms Br1, O2 and Cl1 are located 0.007 (1), 0.012 (4) and 0.024 (2) Å, respectively, from the best least-squares planes of the corresponding rings. The phenyl ring subtends a dihedral angle of 10.3 (2)° with the fused ring system. An intramolecular C—H⋯O occurs (Table 1 ▸).

## 3. Supra­molecular features

**Figure 2:**
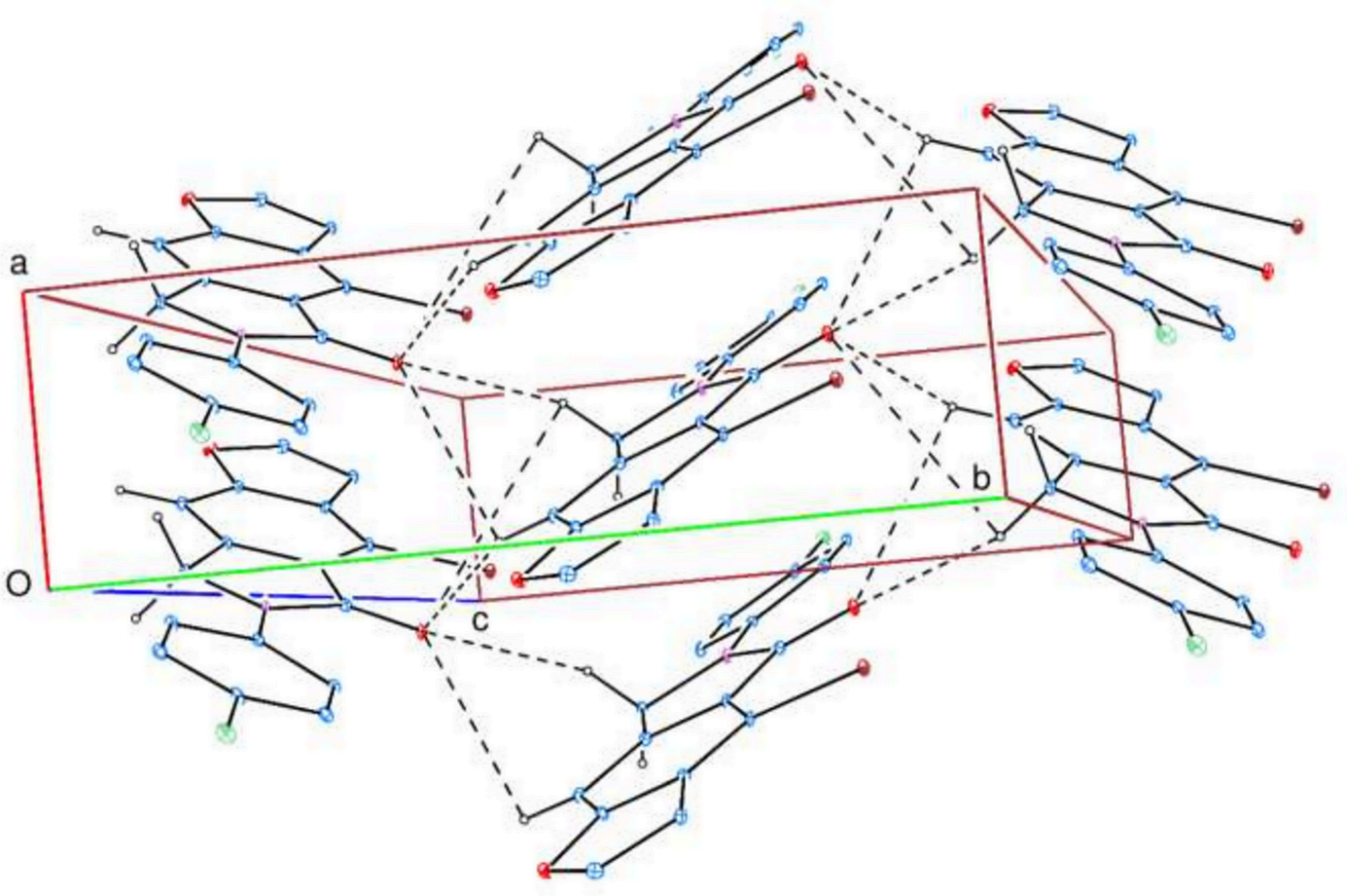
A partial packing diagram of the title compound with C—H⋯O hydrogen bonds shown as dashed lines. H atoms not involved in these inter­actions have been omitted for clarity.

In the crystal, C—H⋯O hydrogen bonds (Table 1 ▸, Fig. 2 ▸) link the mol­ecules into a two-dimensional network nearly parallel to the *ab* plane, enclosing 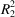
(6), 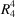
(12), 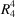
(14), 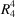
(18) and 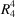
(20) ring motifs (Etter *et al.*, 1990 ▸). π–π inter­actions further consolidate the packing: between *A* rings [centroid-to-centroid distance = 3.919 (3) Å, α = 0.0 (3)° and slippage = 1.971 Å], *B* rings [centroid-to-centroid distance = 3.919 (3) Å, α = 0.0 (3)° and slippage = 1.871 Å], *C* rings [centroid-to-centroid distance = 3.920 (3) Å, α = 0.0 (3)° and slippage = 1.926 Å], *D* rings [centroid-to-centroid distance = 3.919 (3) Å, α = 0.0 (3)° and slippage = 2.022 Å], *A* and *C* rings [centroid-to-centroid distance = 3.642 (3) Å, α = 0.9 (3)° and slippage = 1.289 Å] and *B* and *C* rings [centroid-to-centroid distance = 3.695 (3) Å, α = 0.9 (3)° and slippage = 1.384 Å].

## 4. Hirshfeld surface analysis

**Figure 3:**
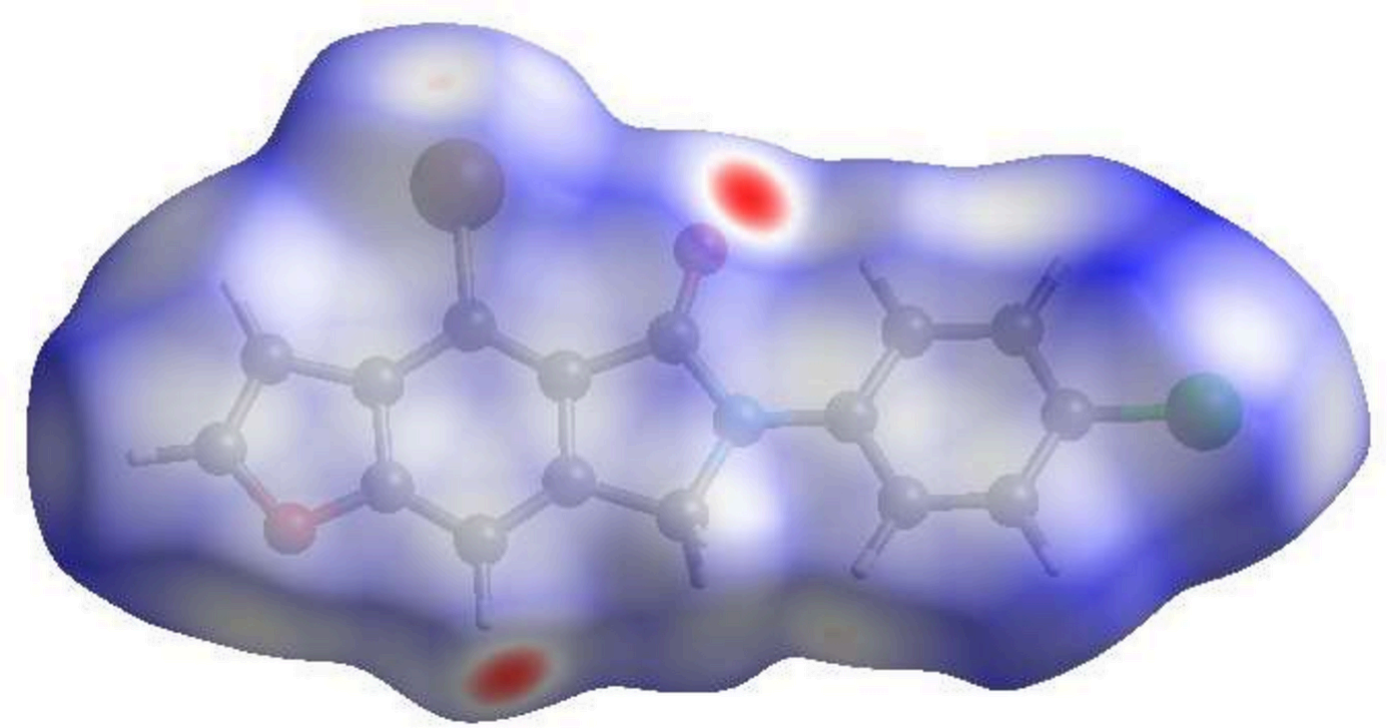
View of the three-dimensional Hirshfeld surface for title mol­ecule plotted over *d*_norm_.

**Figure 4:**
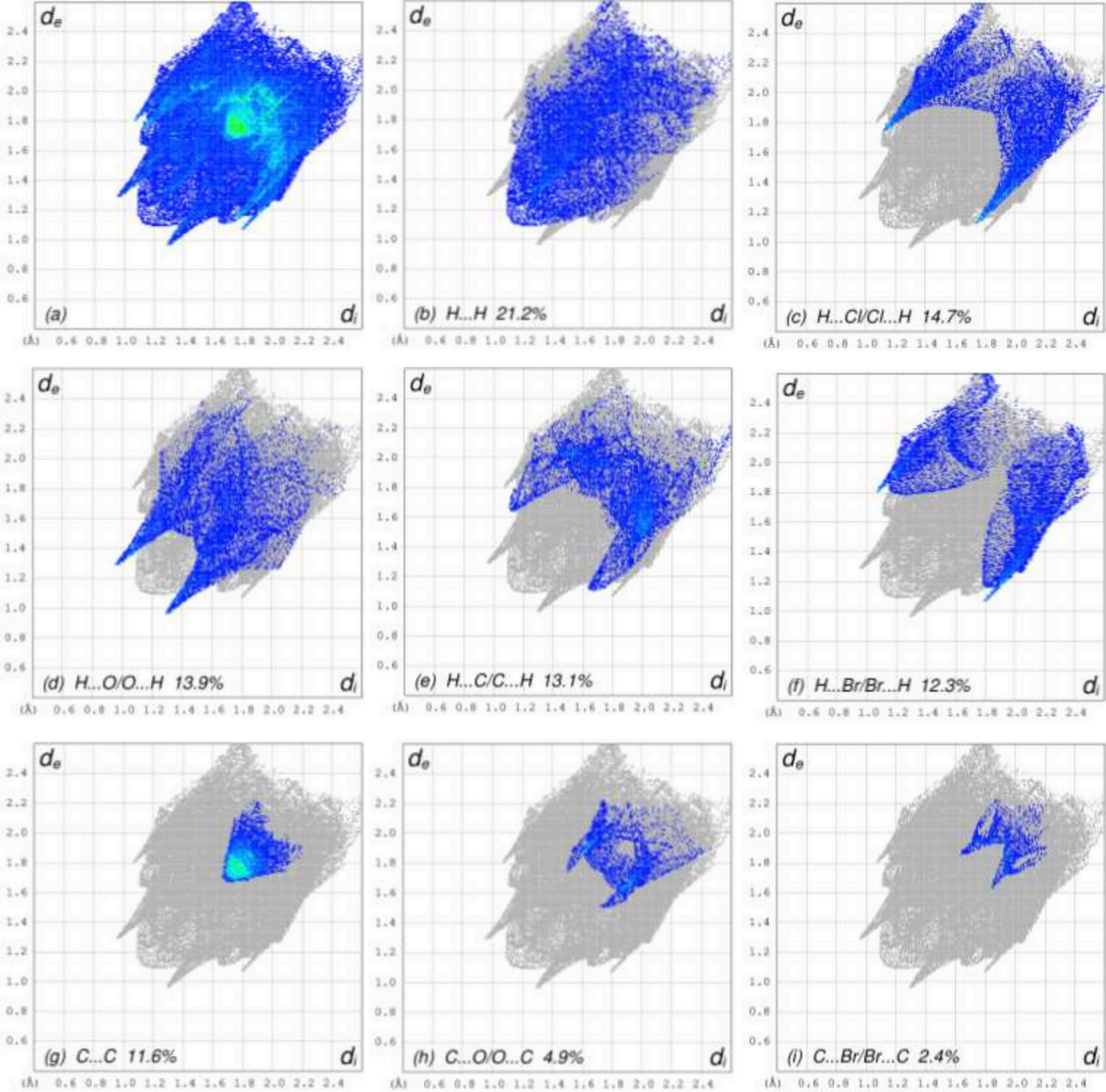
The full two-dimensional fingerprint plots for title mol­ecule, showing (*a*) all inter­actions, and delineated into (*b*) H⋯H, (*c*) H⋯Cl/Cl⋯H, (*d*) H⋯O/O⋯H, (*e*) H⋯C/C⋯H, (*f*) H⋯Br/Br⋯H, (*g*) C⋯C, (*h*) C⋯O/O⋯C, (*i*) C⋯Br/Br⋯C, (*j*) H⋯N/N⋯H, (*k*) Cl⋯Br/Br⋯Cl, (*l*) C⋯Cl/Cl⋯C, (*m*) Cl⋯Cl, (*n*) Br⋯Br, (*o*) C⋯N/N⋯C and (*p*) O⋯O inter­actions. The *d*_i_ and *d*_e_ values are the closest inter­nal and external distances (in Å) from given points on the Hirshfeld surface.

To visualize the inter­molecular inter­actions, a Hirshfeld surface (HS) analysis was carried out using *Crystal Explorer 17.5* (Spackman *et al.*, 2021 ▸). In the HS plotted over *d*_norm_ (Fig. 3 ▸), the contact distances equal, shorter and longer with respect to the sum of van der Waals radii are shown in white, red and blue, respectively. According to the two-dimensional fingerprint plots, H⋯H, H⋯Cl/Cl⋯H, H⋯O/O⋯H, H⋯C/C⋯H, H⋯Br/Br⋯H and C⋯C contacts make the most important contributions to the HS (Table 2 ▸, Fig. 4 ▸).

## 5. Synthesis and crystallization

**Figure 5:**
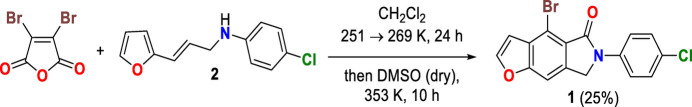
Reaction scheme for obtaining the title compound (**1**).

4-Chloro-*N*-[(2*E*)-3-(furan-2-yl)prop-2-en-1-yl]aniline (0.30 g, 1.3 mmol) (**2**) was dissolved in dry CH_2_Cl_2_ (10 mL) and cooled to 251 K. Di­bromo­maleic anhydride (0.33 g, 1.3 mmol) was added, and the mixture was kept at 269 K for 1 d. The resulting precipitate was filtered off, dissolved in dry DMSO (10 mL), and stirred at 353 K for 10 h. The mixture was poured into water (50 mL), then resulting precipitate was filtered, and washed by water (3 × 3 mL). The product was dried in the air to constant weight to afford compound **1** as light-yellow solid (117.8 mg, 0.33 mmol, 25%, m.p.: 532-533 K). A single crystal suitable for X-ray analysis was obtained from DMSO-*d*_6_ with heating to 353 K and following slow cooling to r.t. The reaction scheme is shown in Fig. 5 ▸). ^1^H NMR (700.2 MHz, DMSO-*d*_6_, 353 K) (*J*, Hz): δ 8.17 (*d*, *J* = 1.4, 1H, H-2-fur­yl), 7.91 (*d*, *J* = 8.6, 2H, H-2,6-C_6_H_4_Cl), 7.85 (*s*, 1H, H-8), 7.47 (*d*, *J* = 8.8, 2H, H-3,5-C_6_H_4_Cl), 7.06 (*br.s*., 1H, H-3-fur­yl), 4.99 (*s*, 2H, H-7) ppm. ^13^C{^1^H}NMR (176.1 MHz, DMSO-*d*_6_, 353 K) δ 164.2 (C=O), 155.7, 147.9, 139.4, 138.0, 129.8, 128.4 (2C, C-2,6-C_6_H_4_Cl), 127.8, 124.0, 120.8 (2C, C-3,5- C_6_H_4_Cl), 110.0, 106.5, 105.5, 48.7 ppm. IR (KBr), ν (cm^−1^) 3732, 3117, 3075, 2927, 1688, 1495, 1385, 1288, 1261, 1065, 825, 758. Analysis calculated for C_16_H_9_BrClNO_2_: C 53.00, H 2.50, N 3.86; found C 52.81, H 2.38, N 3.69.

## 6. Refinement

Crystal data, data collection and structure refinement details are summarized in Table 3 ▸. The C-bond hydrogen-atom positions were calculated geometrically at distances of 0.95 Å (for aromatic CH) and 0.99 Å (for methyl­ene CH) and refined using a riding model by applying the constraint *U*_iso_(H) = 1.2*U*_eq_(C).

## Supplementary Material

Crystal structure: contains datablock(s) I, global. DOI: 10.1107/S2056989025008606/jy2067sup1.cif

Structure factors: contains datablock(s) I. DOI: 10.1107/S2056989025008606/jy2067Isup2.hkl

Supporting information file. DOI: 10.1107/S2056989025008606/jy2067Isup3.cml

CCDC reference: 2492471

Additional supporting information:  crystallographic information; 3D view; checkCIF report

## Figures and Tables

**Table 1:** Hydrogen-bond geometry (Å, °)

*D*—H⋯*A*	*D*—H	H⋯*A*	*D*⋯*A*	*D*—H⋯*A*
C7—H7*B*⋯O2^i^	0.99	2.48	3.387 (7)	152
C8—H8*A*⋯O2^iii^	0.95	2.39	3.267 (7)	154
C12—H12*A*⋯O2	0.95	2.22	2.844 (6)	122

Symmetry codes: (i) 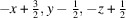; (iii) 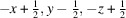.

**Table 2:** Selected interatomic distances (Å)

Br1⋯O2	3.204 (4)	O2⋯H8*A*^ii^	2.39
C16⋯Br1^i^	3.424 (6)	C5⋯H12*A*	2.71
H16*A*⋯Br1^i^	3.03	C7⋯H16*A*	2.46
Br1⋯H16*A*^ii^	3.02	C16⋯H7*A*	2.72
O1⋯C13^iii^	3.218 (7)	C16⋯H7*B*	2.90
O2⋯C12	2.844 (6)	H7*A*⋯H16*A*	2.16
H7*B*⋯O2^i^	2.48		

Symmetry codes: (i) 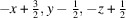; (ii) 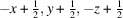; (iii) 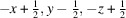.

**Table 3:** Experimental details

Crystal data	
Chemical formula	C_16_H_9_BrClNO_2_
*M*_r_	362.60
Crystal system, space group	Monoclinic, *P*2_1_/*n*
Temperature (K)	100
*a*, *b*, *c* (Å)	3.9194 (7), 12.594 (2), 26.858 (4)
β (°)	91.083 (6)
*V* (Å^3^)	1325.5 (4)
*Z*	4
Radiation type	Mo *K*α
μ (mm^−1^)	3.31
Crystal size (mm)	0.50 × 0.08 × 0.02

Data collection	
Diffractometer	Bruker *KAPPA* APEXII area-detector diffractometer
Absorption correction	Multi-scan (*SADABS*; Krause *et al.*, 2015 ▸)
*T*_min_, *T*_max_	0.556, 1.000
No. of measured, independent and observed [*I* > 2σ(*I*)] reflections	12749, 2992, 1967
*R*_int_	0.132
(sin θ/λ)_max_ (Å^−1^)	0.650

Refinement	
*R*[*F*^2^ > 2σ(*F*^2^)], *wR*(*F*^2^), *S*	0.062, 0.144, 1.04
No. of reflections	2992
No. of parameters	190
H-atom treatment	H-atom parameters constrained
Δρ_max_, Δρ_min_ (e Å^−3^)	1.01, −0.99

Computer programs: *APEX3* and *SAINT* (Bruker, 2018 ▸), *SHELXT* (Sheldrick, 2015*a* ▸), *SHELXL2018/3* (Sheldrick, 2015*b* ▸), *ORTEP-3 for Windows* and *WinGX* publication routines (Farrugia, 2012 ▸) and *PLATON* (Spek, 2020 ▸).

## supplementary crystallographic information

### 4-Bromo-6-(4-chlorophenyl)-6,7-dihydro-5H-furo[2,3-f]isoindol-5-one Crystal data

**Table:**

C_16_H_9_BrClNO_2_	*F*(000) = 720
*M**_r_* = 362.60	*D*_x_ = 1.817 Mg m^−^^3^
Monoclinic, *P*2_1_/*n*	Mo *K*α radiation, λ = 0.71073 Å
*a* = 3.9194 (7) Å	Cell parameters from 1710 reflections
*b* = 12.594 (2) Å	θ = 3.0–28.1°
*c* = 26.858 (4) Å	µ = 3.31 mm^−^^1^
β = 91.083 (6)°	*T* = 100 K
*V* = 1325.5 (4) Å^3^	Plate, colourless
*Z* = 4	0.50 × 0.08 × 0.02 mm

### 4-Bromo-6-(4-chlorophenyl)-6,7-dihydro-5H-furo[2,3-f]isoindol-5-one Data collection

**Table:**

Bruker KAPPA APEXII area-detector diffractometer	1967 reflections with *I* > 2σ(*I*)
φ and ω scans	*R*_int_ = 0.132
Absorption correction: multi-scan (SADABS; Krause *et al.*, 2015)	θ_max_ = 27.5°, θ_min_ = 4.1°
*T*_min_ = 0.556, *T*_max_ = 1.000	*h* = −5→4
12749 measured reflections	*k* = −16→16
2992 independent reflections	*l* = −34→34

### 4-Bromo-6-(4-chlorophenyl)-6,7-dihydro-5H-furo[2,3-f]isoindol-5-one Refinement

**Table:**

Refinement on *F*^2^	0 restraints
Least-squares matrix: full	Hydrogen site location: inferred from neighbouring sites
*R*[*F*^2^ > 2σ(*F*^2^)] = 0.062	H-atom parameters constrained
*wR*(*F*^2^) = 0.144	*w* = 1/[σ^2^(*F*_o_^2^) + 0.5333*P*] where *P* = (*F*_o_^2^ + 2*F*_c_^2^)/3
*S* = 1.04	(Δ/σ)_max_ < 0.001
2992 reflections	Δρ_max_ = 1.01 e Å^−^^3^
190 parameters	Δρ_min_ = −0.99 e Å^−^^3^

### 4-Bromo-6-(4-chlorophenyl)-6,7-dihydro-5H-furo[2,3-f]isoindol-5-one Special details

**Table:**

Geometry. All esds (except the esd in the dihedral angle between two l.s. planes) are estimated using the full covariance matrix. The cell esds are taken into account individually in the estimation of esds in distances, angles and torsion angles; correlations between esds in cell parameters are only used when they are defined by crystal symmetry. An approximate (isotropic) treatment of cell esds is used for estimating esds involving l.s. planes.

### 4-Bromo-6-(4-chlorophenyl)-6,7-dihydro-5H-furo[2,3-f]isoindol-5-one Fractional atomic coordinates and isotropic or equivalent isotropic displacement parameters (Å^2^)

**Table:**

	*x*	*y*	*z*	*U*_iso_*/*U*_eq_	
Br1	0.49744 (14)	0.80459 (5)	0.13637 (2)	0.02236 (18)	
Cl1	1.0284 (4)	0.67377 (13)	0.49698 (5)	0.0357 (4)	
O1	−0.0949 (10)	0.4518 (3)	0.10290 (13)	0.0254 (9)	
O2	0.7205 (10)	0.7699 (3)	0.25059 (13)	0.0228 (9)	
N6	0.5883 (11)	0.6079 (4)	0.28682 (15)	0.0191 (10)	
C2	−0.0979 (15)	0.5213 (5)	0.06345 (19)	0.0265 (14)	
H2A	−0.200214	0.505380	0.031985	0.032*	
C3	0.0575 (14)	0.6138 (5)	0.07363 (19)	0.0219 (13)	
H3A	0.079676	0.673342	0.052237	0.026*	
C3A	0.1838 (14)	0.6031 (5)	0.12427 (19)	0.0210 (12)	
C4	0.3565 (14)	0.6695 (4)	0.15773 (19)	0.0191 (12)	
C4A	0.4241 (14)	0.6307 (4)	0.20511 (18)	0.0197 (12)	
C5	0.5962 (14)	0.6812 (5)	0.24847 (19)	0.0217 (13)	
C7A	0.3151 (14)	0.5294 (5)	0.21916 (18)	0.0210 (12)	
C7	0.4199 (14)	0.5088 (4)	0.27276 (18)	0.0202 (12)	
H7A	0.218906	0.495166	0.293605	0.024*	
H7B	0.578659	0.447864	0.275526	0.024*	
C8A	0.0764 (14)	0.5035 (5)	0.14046 (19)	0.0216 (13)	
C8	0.1392 (14)	0.4615 (5)	0.18745 (19)	0.0236 (13)	
H8A	0.068007	0.392468	0.196964	0.028*	
C11	0.7085 (14)	0.6249 (5)	0.33650 (19)	0.0203 (12)	
C12	0.8993 (14)	0.7137 (4)	0.35026 (19)	0.0214 (12)	
H12A	0.964013	0.763946	0.325817	0.026*	
C13	0.9952 (15)	0.7287 (5)	0.4000 (2)	0.0258 (13)	
H13A	1.121382	0.789909	0.409754	0.031*	
C14	0.9054 (15)	0.6538 (5)	0.43502 (19)	0.0245 (13)	
C15	0.7284 (15)	0.5643 (5)	0.4216 (2)	0.0272 (14)	
H15A	0.675257	0.512542	0.445981	0.033*	
C16	0.6270 (14)	0.5492 (5)	0.37244 (19)	0.0229 (13)	
H16A	0.501947	0.487489	0.363146	0.027*	

### 4-Bromo-6-(4-chlorophenyl)-6,7-dihydro-5H-furo[2,3-f]isoindol-5-one Atomic displacement parameters (Å^2^)

**Table:**

	*U*^11^	*U*^22^	*U*^33^	*U*^12^	*U*^13^	*U*^23^
Br1	0.0309 (4)	0.0180 (4)	0.0183 (3)	−0.0014 (2)	0.00298 (19)	0.0033 (2)
Cl1	0.0482 (11)	0.0405 (12)	0.0181 (7)	0.0113 (7)	−0.0062 (6)	−0.0066 (6)
O1	0.029 (3)	0.028 (3)	0.019 (2)	−0.0065 (17)	0.0006 (16)	−0.0044 (17)
O2	0.036 (3)	0.014 (3)	0.0179 (19)	−0.0052 (16)	0.0019 (15)	0.0007 (15)
N6	0.029 (3)	0.012 (3)	0.016 (2)	−0.0013 (18)	0.0023 (18)	0.0014 (18)
C2	0.030 (4)	0.036 (4)	0.013 (3)	−0.001 (3)	0.000 (2)	0.001 (2)
C3	0.029 (4)	0.018 (4)	0.019 (3)	0.001 (2)	0.005 (2)	0.001 (2)
C3A	0.022 (4)	0.021 (4)	0.020 (3)	−0.001 (2)	0.007 (2)	0.001 (2)
C4	0.028 (4)	0.011 (3)	0.018 (3)	0.003 (2)	0.006 (2)	0.000 (2)
C4A	0.025 (4)	0.017 (4)	0.018 (3)	−0.001 (2)	0.008 (2)	0.000 (2)
C5	0.026 (4)	0.021 (4)	0.018 (3)	0.004 (2)	0.005 (2)	−0.002 (2)
C7A	0.025 (4)	0.022 (4)	0.016 (3)	0.002 (2)	0.002 (2)	0.000 (2)
C7	0.032 (4)	0.015 (4)	0.013 (2)	−0.002 (2)	0.002 (2)	−0.001 (2)
C8A	0.023 (4)	0.022 (4)	0.020 (3)	−0.002 (2)	0.000 (2)	−0.003 (2)
C8	0.028 (4)	0.020 (4)	0.023 (3)	0.003 (2)	0.004 (2)	−0.002 (2)
C11	0.022 (4)	0.020 (4)	0.019 (3)	0.005 (2)	0.001 (2)	0.000 (2)
C12	0.027 (4)	0.020 (4)	0.017 (3)	0.004 (2)	0.002 (2)	0.000 (2)
C13	0.030 (4)	0.019 (4)	0.028 (3)	0.006 (2)	−0.005 (2)	−0.005 (2)
C14	0.036 (4)	0.019 (4)	0.018 (3)	0.012 (2)	−0.004 (2)	−0.003 (2)
C15	0.031 (4)	0.028 (4)	0.022 (3)	0.008 (3)	0.004 (2)	0.006 (3)
C16	0.033 (4)	0.019 (4)	0.016 (3)	0.004 (2)	0.001 (2)	−0.003 (2)

### 4-Bromo-6-(4-chlorophenyl)-6,7-dihydro-5H-furo[2,3-f]isoindol-5-one Geometric parameters (Å, º)

**Table:**

Br1—C4	1.882 (5)	C7A—C8	1.381 (7)
Cl1—C14	1.742 (5)	C7A—C7	1.512 (7)
O1—C8A	1.367 (6)	C7—H7A	0.9900
O1—C2	1.375 (6)	C7—H7B	0.9900
O2—C5	1.219 (6)	C8A—C8	1.386 (7)
N6—C5	1.384 (7)	C8—H8A	0.9500
N6—C11	1.423 (6)	C11—C12	1.392 (8)
N6—C7	1.458 (7)	C11—C16	1.397 (7)
C2—C3	1.340 (8)	C12—C13	1.394 (7)
C2—H2A	0.9500	C12—H12A	0.9500
C3—C3A	1.445 (7)	C13—C14	1.383 (8)
C3—H3A	0.9500	C13—H13A	0.9500
C3A—C4	1.393 (7)	C14—C15	1.368 (8)
C3A—C8A	1.395 (8)	C15—C16	1.385 (7)
C4—C4A	1.384 (7)	C15—H15A	0.9500
C4A—C7A	1.399 (8)	C16—H16A	0.9500
C4A—C5	1.479 (7)		
			
Br1···O2	3.204 (4)	H7B···O2^i^	2.48
C16···Br1^i^	3.424 (6)	O2···H8A^ii^	2.39
H16A···Br1^i^	3.03	C5···H12A	2.71
Br1···H16A^ii^	3.02	C7···H16A	2.46
O1···C13^iii^	3.218 (7)	C16···H7A	2.72
O2···C12	2.844 (6)	C16···H7B	2.90
O2···H12A	2.23	H7A···H16A	2.16
			
C8A—O1—C2	105.2 (4)	N6—C7—H7B	111.3
C5—N6—C11	125.9 (5)	C7A—C7—H7B	111.3
C5—N6—C7	113.3 (4)	H7A—C7—H7B	109.2
C11—N6—C7	120.8 (4)	O1—C8A—C8	124.5 (5)
C3—C2—O1	113.5 (5)	O1—C8A—C3A	110.2 (5)
C3—C2—H2A	123.2	C8—C8A—C3A	125.3 (5)
O1—C2—H2A	123.2	C7A—C8—C8A	113.8 (5)
C2—C3—C3A	104.9 (5)	C7A—C8—H8A	123.1
C2—C3—H3A	127.5	C8A—C8—H8A	123.1
C3A—C3—H3A	127.5	C12—C11—C16	119.5 (5)
C4—C3A—C8A	119.0 (5)	C12—C11—N6	122.5 (5)
C4—C3A—C3	134.8 (5)	C16—C11—N6	118.0 (5)
C8A—C3A—C3	106.1 (5)	C11—C12—C13	119.8 (5)
C4A—C4—C3A	117.6 (5)	C11—C12—H12A	120.1
C4A—C4—Br1	123.2 (4)	C13—C12—H12A	120.1
C3A—C4—Br1	119.2 (4)	C14—C13—C12	119.5 (6)
C4—C4A—C7A	121.0 (5)	C14—C13—H13A	120.3
C4—C4A—C5	130.4 (5)	C12—C13—H13A	120.3
C7A—C4A—C5	108.5 (5)	C15—C14—C13	121.1 (5)
O2—C5—N6	126.2 (5)	C15—C14—Cl1	120.0 (5)
O2—C5—C4A	127.4 (5)	C13—C14—Cl1	118.8 (5)
N6—C5—C4A	106.4 (5)	C14—C15—C16	119.9 (5)
C8—C7A—C4A	123.3 (5)	C14—C15—H15A	120.0
C8—C7A—C7	127.2 (5)	C16—C15—H15A	120.0
C4A—C7A—C7	109.5 (4)	C15—C16—C11	120.0 (5)
N6—C7—C7A	102.3 (4)	C15—C16—H16A	120.0
N6—C7—H7A	111.3	C11—C16—H16A	120.0
C7A—C7—H7A	111.3		

Symmetry codes: (i) −*x*+3/2, *y*−1/2, −*z*+1/2; (ii) −*x*+1/2, *y*+1/2, −*z*+1/2; (iii) −*x*+1/2, *y*−1/2, −*z*+1/2.

### 4-Bromo-6-(4-chlorophenyl)-6,7-dihydro-5H-furo[2,3-f]isoindol-5-one Hydrogen-bond geometry (Å, º)

**Table:**

*D*—H···*A*	*D*—H	H···*A*	*D*···*A*	*D*—H···*A*
C7—H7*B*···O2^i^	0.99	2.48	3.387 (7)	152
C8—H8*A*···O2^iii^	0.95	2.39	3.267 (7)	154
C12—H12*A*···O2	0.95	2.22	2.844 (6)	122

Symmetry codes: (i) −*x*+3/2, *y*−1/2, −*z*+1/2; (iii) −*x*+1/2, *y*−1/2, −*z*+1/2.
